# Immature Teratoma of the Palm: A Rare Site of Presentation

**DOI:** 10.7759/cureus.10738

**Published:** 2020-09-30

**Authors:** Jyoti Rajpoot, Sufian Zaheer, Sugandha Sugandha, Sunil Ranga

**Affiliations:** 1 Department of Pathology, Vardhman Mahavir Medical College and Safdarjung Hospital, New Delhi, IND

**Keywords:** teratoma, palm, extragonadal, germ cells tumor

## Abstract

Extragonadal teratomas in adults are an extremely rare entity. Teratomas may be mature or immature and are characterized by their midline presentation. We are presenting here a case report of immature teratoma of the palm in a 35-year-old female. The patient developed a recurrent swelling of the palm from which a wedge biopsy was taken and sent for histopathological examination. A diagnosis of “immature teratoma, grade 2” was made, whose margins were compromised. Later on ultrasonography (USG) abdomen and pelvis and whole-body magnetic resonance imaging (MRI) showed no significant findings ruling out the possibility of an occult primary. MRI palm showed a well-defined cystic lesion with loculations suggestive of teratoma. Re-exploration was advised to achieve free margins.

## Introduction

A teratoma, a type of germ cell tumor, maybe gonadal or extragonadal. Primary extragonadal germ cell tumors as a whole are extremely rare in incidence. Primary extragonadal germ cell tumors are characterized by their midline location in the body [1]. The pathogenesis of these tumors is poorly understood and controversial. A possible explanation for their origin in the midline is the development from a sequestered germ cell during embryogenesis [2]. Also, a second theory has been suggested that possibly mediastinal extragonadal germ cell tumors may be originating from the reverse migration of occult carcinoma in situ lesions in the gonad [3]. Among these various extragonadal sites like the craniofacial region, sacrococcygeal region, cervical region, the midline of the central nervous system, mediastinum, and retroperitoneum, the mediastinum is by far the most common site comprising 50% to 70% of extragonadal teratomas [4]. In the pediatric age group, the sacrococcygeal region is the most common site [5]. The only known predisposing risk factor is Klinefelter syndrome (47 XXY). The exact incidence is unknown but individuals with this syndrome have increased incidence of mediastinal or mixed germ cell tumors [6]. Individual case reports have been published for the thyroid, stomach, liver, heart, pleura, pharynx, vagina, prostate, and subcutaneous tissue [7]. We are presenting here a rare case report, probably the first one in the literature, to the best of our knowledge, of a primary immature teratoma arising in the palm without evidence of involvement of any other organ on thorough clinico-radiological evaluation.

## Case presentation

**Figure 1:**
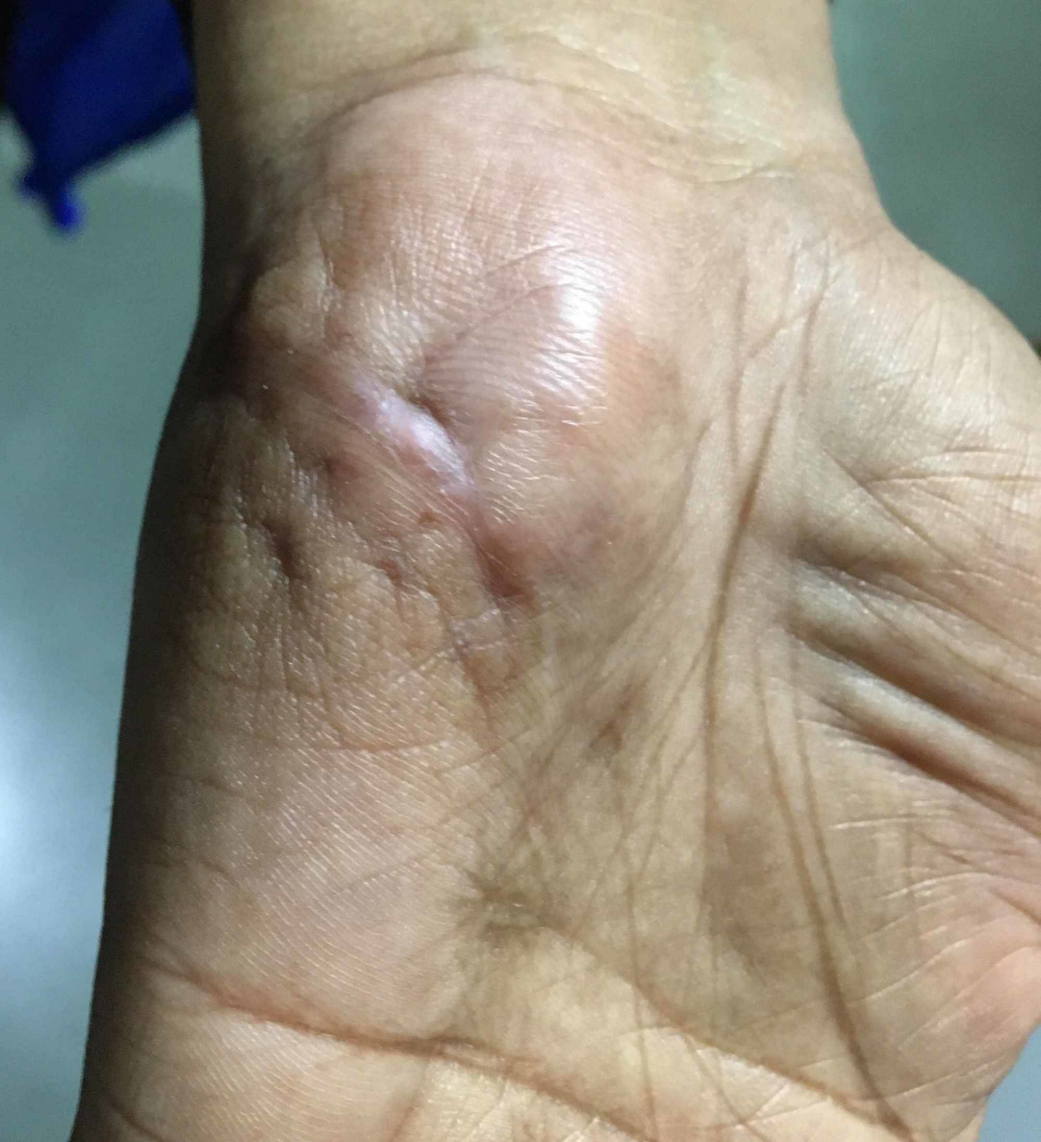
Ill-defined swelling over the palm, a scar mark can be seen

A 35-year-old female presented to our hospital with a complaint of a recurrent ill-defined nodular swelling measuring 3×2 cm over the palm for the past one month (Figure 1).

**Figure 2:**
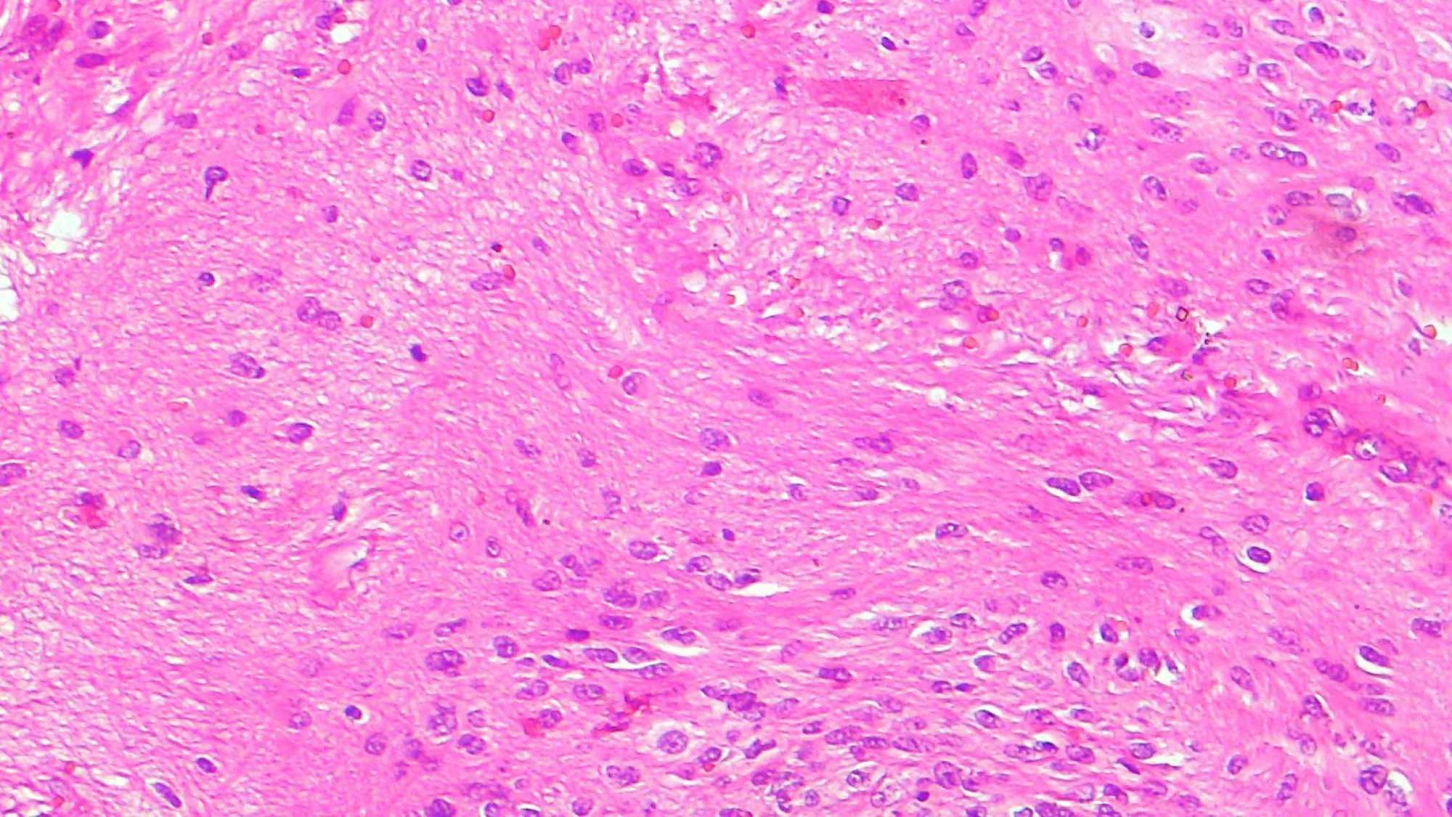
H&E shows mature glial cells in the neurofibrillary stroma H&E: hematoxylin and eosin

**Figure 3:**
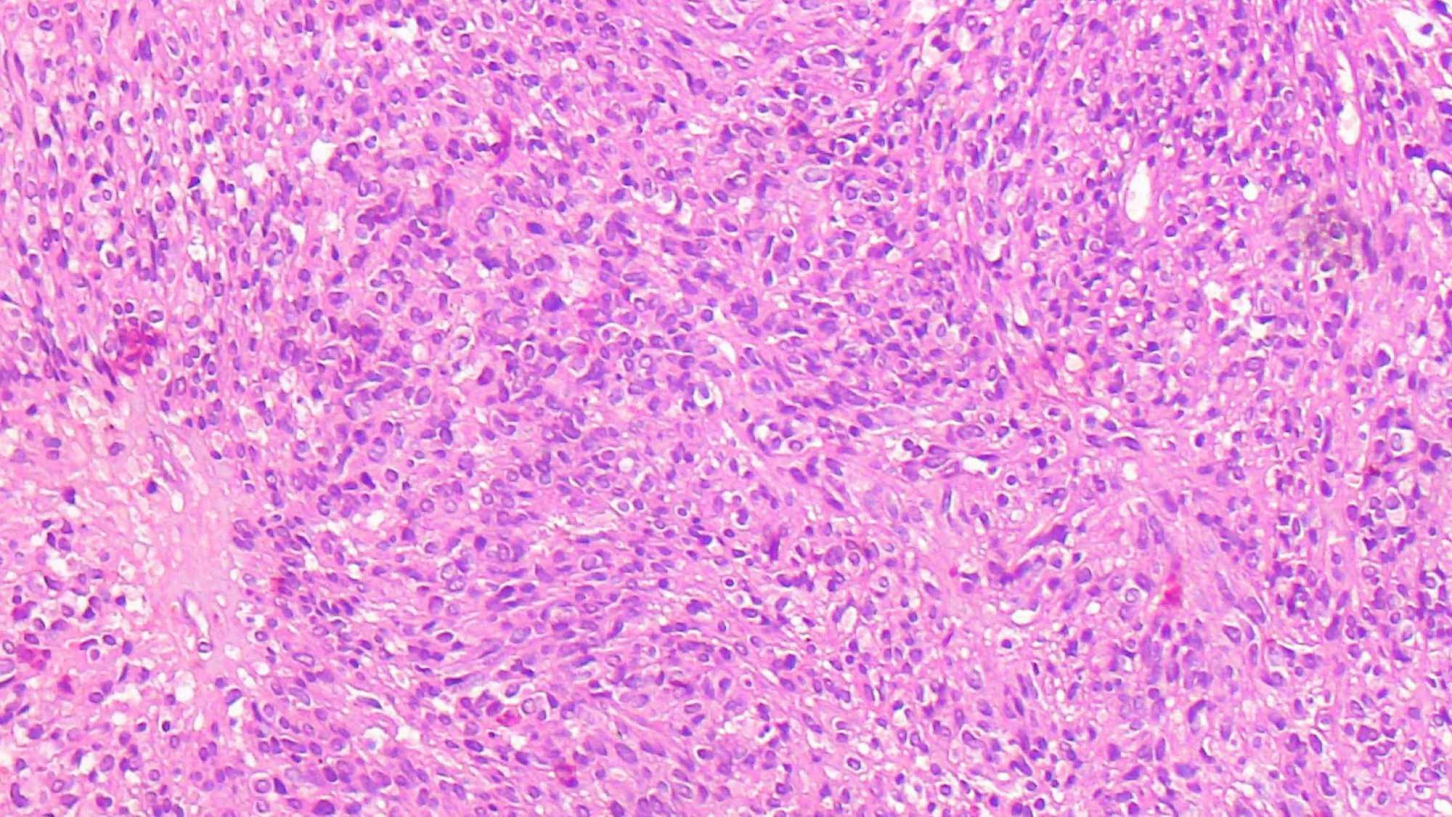
Immature neuroepithelial tissue showing dark hyperchromatic nuclei

The overlying skin was bluish in coloration and showed the scar of the previous surgery. The patient gave a history of recent surgical excision of a soft tissue mass at the same site measuring around 4X3 cm three months ago. Outside the suggestion of the possibility of a germ cell tumor, no immunohistochemistry was performed. We received all the paraffin blocks for review. On histopathology, there were predominantly mature glial cells (Figure 2), immature neural tissue (Figure 3), and glandular tissue, along with cartilage and skin tissue.

**Figure 4:**
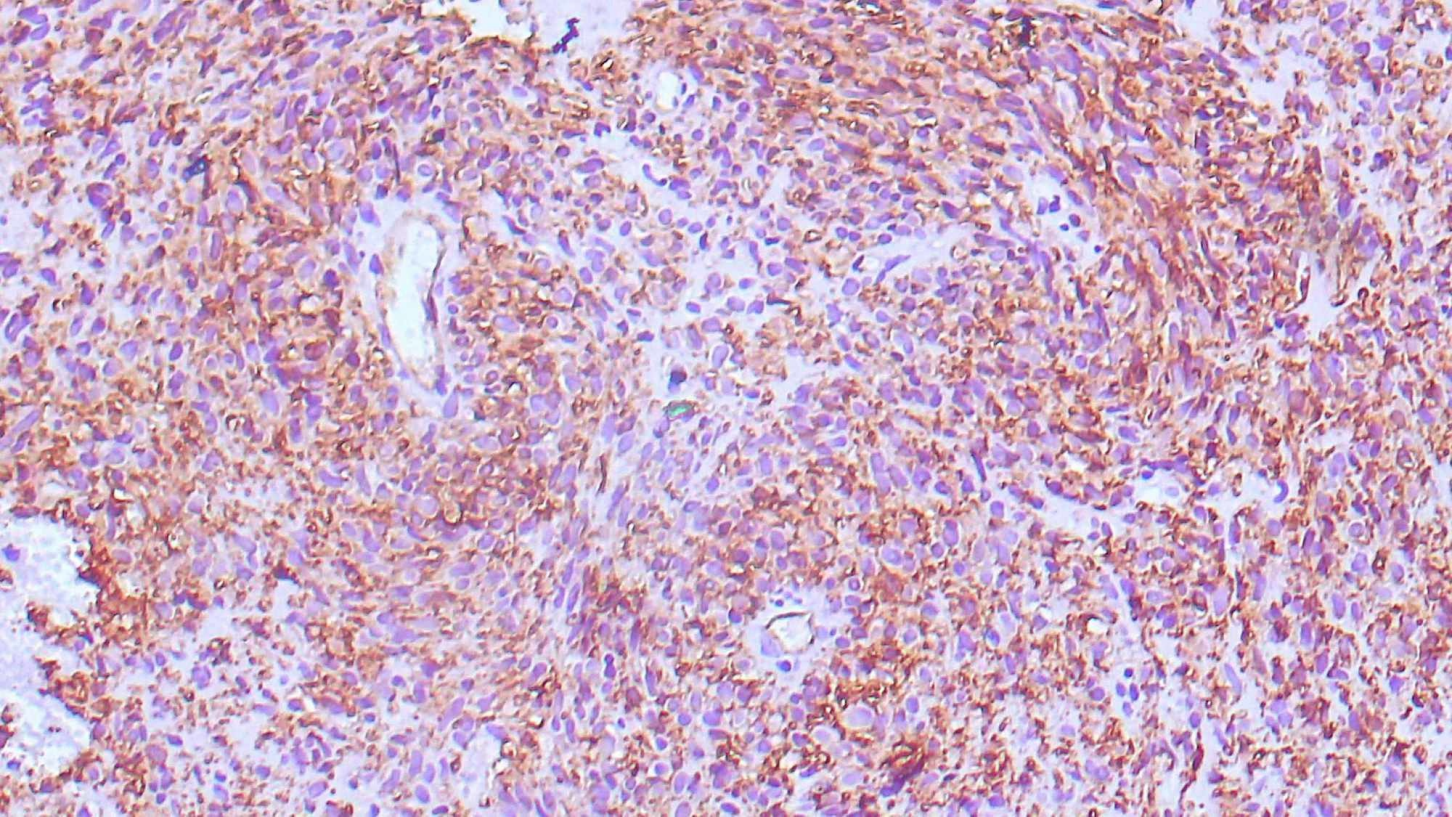
Immature neural tissue showing positivity for NSE NSE: neuron-specific enolase

One of the resected margins was involved by the tumor. On immunohistochemistry, mature neural tissue was positive for glial fibrillary acidic protein (GFAP) and immature neural tissue was positive for neuron-specific enolase (NSE) (Figure 4).

**Figure 5:**
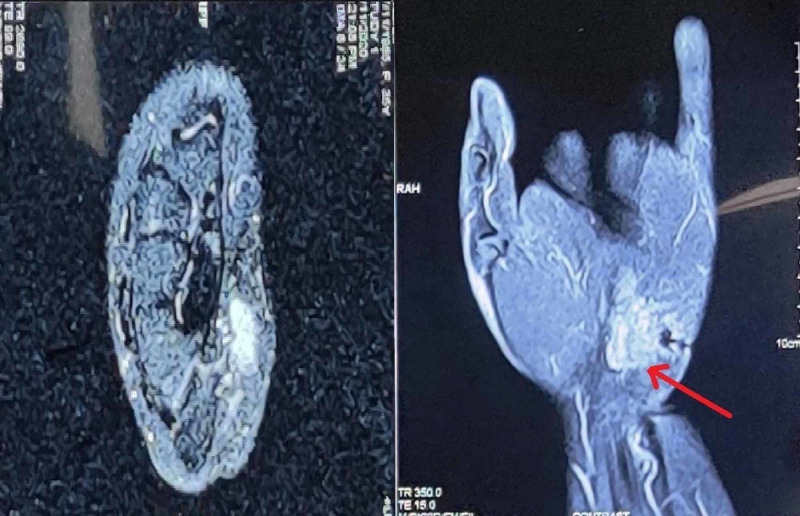
MRI left hand showing a well-defined cystic lesion with internal loculations MRI: magnetic resonance imaging

Grading was done on the basis of the content of immature neuroepithelium. Hormonal investigations were done. Alpha-fetoprotein (7.1 ng/ml) (Normal range: <10 ng/mL) and beta-human chorionic gonadotropin (1.4 mIU/ml) (Normal range: <10.0 mIU/mL) levels were normal. Ultrasonography abdomen and pelvis showed bilateral normal adnexa with no significant lymphadenopathy. Uterus showed multiple fibroids. Whole-body MRI showed no significant abnormality except MRI hand. MRI left hand (Figure 5) showed a well-defined cystic lesion on the volar aspect of the hand in the hypothenar region located within the subcutaneous plane, with internal loculations suggestive a teratoma.

A wedge biopsy was done from the present swelling, which showed similar morphological features, including immature neural tissue, which was positive for NSE. After clinico-radiological correlation, a final diagnosis of a primary immature teratoma, grade 2, of the palm was given. A repeat surgical excision of the swelling with the restoration of clear margins was advised.

## Discussion

Broadly, teratomas are classified as mature and immature. Mature teratomas are considered benign tumors. They may be cystic or solid and consist of well-differentiated adult tissues from one or more germ cell layers [8]. Endodermal germ cells show intestinal glands/mucosa, respiratory epithelium, thyroid tissue, and renal tissue. Ectodermal germ cell derivatives are usually skin, sebaceous glands, sweat glands, neural tissue, and dental tissue. The mesodermal germ layer’s structures comprise adipose tissue, bone, cartilage, muscle, and loose connective tissue. Immature teratomas are malignant tumors. They show the presence of mature elements from one or more cell layers along with immature elements, which are mostly neuroectodermal in origin followed by mesodermal elements [9]. These immature neuroectodermal elements are monomorphic in appearance having scant cytoplasm and dark nuclei. There may be true rosettes, pseudo-rosettes, and tubule formation. Typical and atypical mitoses are frequent. Mesodermal immature elements consist of sheets of spindle cells with hyperchromatic nuclei. Immature cartilage may also be present. This cartilage shows medium-sized chondrocytes with vesicular nuclei and these foci are surrounded by small, immature mesenchymal cells. Immature endodermal elements show primitive glands lined by cells with sub-nuclear and supra-nuclear vacuoles.

Grading of immature teratomas is based on World Health Organization (WHO) 2014 recommendations. Previously, a three-tier grading system was used, which has been modified to “low grade” (Grade 1) and “high grade” (Grade 2 and Grade 3) immature teratoma [10].

Prognosis depends on certain factors like histological type and grade, patient’s age, anatomic location, the status of surgical resection, and metastatic spread [11]. In the pediatric and adolescent age groups, immature teratomas behave as benign lesions with an excellent outcome. In adults, immature teratomas show aggressive behavior with significant metastatic potential. Immunohistochemistry plays a supportive role in the confirmation of histomorphological findings. GFAP stains mature glial fibrils and neural cell bodies. It does not stain primitive neuroepithelial tissue, which stains positive with NSE, CD99, bcl2, and neurofilament [12]. Individual case reports and case series have been published about extragonadal immature teratomas presenting at sites outside midline. Zuquello et al. published a case report of an immature teratoma presenting as a thigh mass and suggested the possibility of it being a secondary to occult gonadal or retroperitoneal primary [13]. Koh et al. reported a case of a malignant teratoma of the proximal humerus in a young girl. Radiology suggested a malignant bone tumor but histopathology was that of an immature bony teratoma. No other abnormal lesion could be identified with positron emission tomography-computed tomography (PET-CT) [14]. Benali et al. described the first and only case of a mixed germ cell tumor in the soft tissue of the right arm [15]. Likewise, cases are reported from the neck, stomach, liver, abdomen, and pelvis [16-18]. In our case, clinicians were suspecting an abscess or inflamed benign cyst. Thorough USG abdomen and pelvis and whole-body MRI showed no other abnormal findings. Also, serological markers were in the low normal range. Considering normal radiological findings and normal serological markers, the possibility of the gonadal or extragonadal primary at the usual sites was ruled out. We searched the literature and could not find any case of immature teratoma reported from the palm, so this being the first case reported of this kind. An extragonadal teratoma mostly arises in the midline along the path of migration of germ cells [19]. We were not able to explain the origin of teratoma at this site, and more case studies and case reviews need to be reported at these unusual sites to accurately understand the pathogenesis of such presentations. Management includes complete surgical excision without adjuvant chemo or radiotherapy; still, the use of adjuvant therapies is controversial in high-grade teratomas [20].

## Conclusions

We could not find any paper published on an immature teratoma in the palm so our case is extremely rare and unique in presentation. In conclusion, an extragonadal teratoma may develop in sites other than the midline so it should be considered in the differential diagnosis by clinicians. More cases like this presentation need to be reported after proper workup to understand the etiopathogenesis of extragonadal teratomas well.

## References

[1] Gao Y, Jiang J, Liu Q (2015). Extragonadal malignant germ cell tumors: a clinicopathological and immunohistochemical analysis of 48 cases at a single Chinese institution. Int J Clin Exp Pathol.

[2] Ashley DJB (1973). Origin of teratomas. Cancer.

[3] Böhle A, Studer UE, Sonntag RW, Scheidegger JR (1986). Primary or secondary extragonadal germ cell tumors?. J Urol.

[4] Shinagare AB, Jagannathan JP, Ramaiya NH, Hall MN, Van den Abbeele AD (2010). Adult extragonadal germ cell tumours. Am J Roentgenol.

[5] Heerema‐McKenney A, Harrison MR, Bratton B, Farrell J, Zaloudek C (2005). Congenital teratoma. A clinicopathologic study of 22 fetal and neonatal tumors. Am J Surg Pathol.

[6] Williams LA, Pankratz N, Lane J (2018). Klinefelter syndrome in males with germ cell tumors: a report from the Children's Oncology Group. Cancer.

[7] Sachveda K (2020). Extragonadal germ cell tumors. Medscape Medical Reference. https://emedicine.medscape.com/article/278174-overview.

[8] Ronchi A, Cozzolino I, Montella M (2019). Extragonadal germ cell tumors: not just a matter of location. A review about clinical, molecular and pathological features. Cancer Med.

[9] O'Donovan EJ, Thway K, Moskovic EC (2014). Extragonadal teratomas of the adult abdomen and pelvis: a pictorial review. Br J Radiol.

[10] WHO WHO (2014). WHO Classification of Tumours of Female Reproductive Organs. WHO Classification of Tumours, 4th Edition, Volume 6. Lyon: IARC.

[11] Moran CA, Suster S (1997). Primary germ cell tumors of the mediastinum. I. Analysis of 322 cases with special emphasis on teratomatous lesions and a proposal for histopathologic classification and clinical staging. Cancer.

[12] Vance RP, Geisinger KR, Randall MB, Marshall RB (1988). Immature neural elements in immature teratomas. An immunohistochemical and ultrastructural study. Am J Clin Pathol.

[13] Zuquello RÁ, Tagliari G, Bagatini R (2016). Immature teratoma presenting as a soft-tissue mass with no evidence of other sites of involvement: a case report. Diagn Pathol.

[14] Koh JS, Park JH, Kang CH (2009). A primary extragonadal teratoma of the proximal humerus. J Korean Med Sci.

[15] Benali HA, Lalya I, Allaoui M (2012). Extragonadal mixed germ cell tumor of the right arm: description of the first case in the literature. World J Surg Oncol.

[16] Aihole JS, Babu M N, Jadhav V, Javaregowda D (2017). Gastric teratoma: an unusual presentation and location. Indian J Med Paediatr Oncol.

[17] Tapper D, Lack EE (1983). Teratomas in infancy and childhood. A 54-year experience at the Children’s Hospital Medical Center. Ann Surg.

[18] Yang DM, Jung DH, Kim H, Kang JH, Kim SH, Kim JH, Hwang HY (2004). Retroperitoneal cystic masses: CT, clinical, and pathologic findings and literature review. Radiographics.

[19] Batsakis JG, el-Naggar AK, Luna MA (1995). Teratomas of the head and neck with emphasis on malignancy. Ann Otol Rhinol Laryngol.

[20] Marina NM, Cushing B, Giller R (1999). Complete surgical excision is effective treatment for children with immature teratomas with or without malignant elements: a Pediatric Oncology Group/Children's Cancer Group Intergroup Study. J Clin Oncol.

